# Arthur's System of Treatment for the Prevention of Decay of the Teeth

**Published:** 1872-04

**Authors:** O. A. Jarvis

**Affiliations:** New York.


### ARTICLE IX.

### *Dr. Arthur's System of Treatment for the Prevention of Decay of the Teeth.*

BY O. A. JARVIS, D. D. S., NEW YORK.

It must be a real pleasure to every right-minded dentist to receive any creditable addition to dental literature. Its paucity is felt and deplored. Yet it is well not to be in haste to add text, standard, or popular works on dentistry until principles are better understood and practice better defined.

We question the wisdom of advocating with so much boldness a practice so at variance with nature, and receiving such doubtful support from results in practice.

Whatever may be the influence of this little book upon the practice of the profession, it is certainly calculated to weaken the confidence of the people in dentists and dental operations.

Let us examine the main idea of this little book,—viz., artificial separation for the prevention of decay. This idea has been forced upon the consideration of every man who has ever filled teeth, or tried to save them by any means; and it has been resorted to in every man's practice to the fullest extent allowed by judgment and experience. We will study this book with all diligence to learn how to do better. We are thankful for the book, for what it teaches, —especially for what it suggests. Some will find a ready apology for their own shortcomings in the general failure which the author affirms; others will be stirred to deeper thought and more earnest efforts for a fuller measure of success.

The proposed plan is not confined to certain forms or classes of teeth, but is put forward as a "new method,"—a substitute for filling.

The first point I make is, that the separation answers the desired end only in so far as it renders the surfaces self-cleansing; that is, the food, in the act of mastication, enters the first V, which is naturally formed by the rounded margins of the grinding surfaces—and by Prof. Arthur's plan is artificially enlarged—is forced past the double apex into the second V, at the cervix, where it lays in contact with the gum. If the space will admit sufficient food, of a somewhat solid character, it will cause pain in the gum, thus necessitating change, which generally secures safety. Then if we add a third V, with apex pointing to the buccal surface, it would seem impossible to any chewing on those teeth, or even to take fluid in the mouth, without changing the contents of those spaces. This is a very common type, such as are represented by Fig. 1. But Prof. Arthur does not leave them even here, without the point of bearing unchanged, but requires that the separation be "thorough, free from the grinding surface to the gum. "p. 155.

Let us merely name some of the objections to this treatment:

First. It is unnatural,—it does not harmonize with the plan of the original artist; and progressive dentists are increasingly inclined to follow the teachings and preserve the types nature gives.

The first or outer V, in its normal condition, is small or restricted, apparently for the purpose of *preventing* the passage of food between the teeth, as well as to furnish the greater extent of grinding surface. Prof. Arthur's proposition is to enlarge that V so that the food, pick, tape, etc., *may* pass freely.

Second. The point of contact in the normal condition of the teeth is almost invariably *single*, and generally more limited in extent than can be secured by artificial separation. The point of bearing should never be removed, or, if removed, it should be restored. Go all around it; restrict or lessen it as much as you please; but if removed, it is an irretriveable injury—there can be no possible compensation for it.

Third. The injury to the gums and process by the pressure of food in the act of mastication, and the violent pressing of the tooth in one direction, and the next instant in the opposite direction; the present pain and resulting periodontitis and probable absorption; the annoyance to the tongue by the artificial angles.

There is positively no weight in the testimony of a few patients to the effect that they experience no inconvenience: we have not yet to learn that people will get used to a mouthful of broken and decayed teeth. But if we are going to appeal to the people for their verdict, we have it already in the universal horror and undying dread with which they have regarded the practice for the past thirty years. It is often alluded to in this book as a prejudice. It is testimony, as sound as the other.

Fourth. The teeth generally do, and may be expected to, shift their position, unless prevented by antagonists. The movement will be in a direction opposite to the slope upon the grinding surface doing the most work. Sometimes it will be lateral, sometimes rotary; often both. In fact, a tooth will not stand alone, independent of companions or antagonists, when subjected to lateral pressure.

It would not seem necessary to inquire if the separation can be maintained when made at the early age required for the prevention of caries, before the teeth had taken their position. See Figs. 28 and 33.

When after separation they come together again, as Prof. Arthur says they "not unfrequently do," (p. 98), and "they are liable to," (p. 156), we are almost sure to have a broader surface in contact, perhaps more than one point, and certainly a narrower space at the cervix, the very place where the space is most needed, and where the very act of coming together produces pressure and consequent absorption,—the difficulties of inspection being thus immeasurably increased. But our author says "cut again," (pp. 98 and 156). Why not as well say "fill again," or repair? If timely, it would be as easy and as sure.

If there is any complication of difficulties to be dreaded, it is such as arises from separations and shifting.

Fifth. Another but slight objection is the fact that exposed dentine always discolors.

Sixth. It is unnecessary; there are two plans, either of which is better. First, the care on the part of the dentist and the patient which Prof. Arthur requires, (p. 159), if timely applied, would probably secure the desired results; second, there is a better plan of separation. Judging from experience in my own mouth and in practice, if my teeth were to be treated by cutting away anywhere for the prevention or arrest of decay, I would prefer it should be done by enlarging the inner or second V, leaving the grinding surfaces intact. This plan would give space where most needed; would more readily reach the point where decay commences; need not endanger the pulp; the surfaces could be as easily inspected; there would be no change of position; there would be no angles and less cut surface; it can be easier done; there would be no inconvenience in mastication; properly done, the gum would probably not change its relation; and finally, it would be quite improbable that so large a quantity of bread and potatoes would be wasted in the mouth of any patient who had passed under the hands of a faithful dentist. This I propose and practice as a substitute for Prof. Arthur's plan for *prevention* and the removal of incipient proximate decay. But let us try again the filling plan, pronounced by Prof. Arthur a failure, as applied to the proximate surfaces of the bicuspids and molars.

Imperfect operations are a more prolific source of failure than all others combined. This a fact which can be demonstrated. To enter into the details at present would be too tedious.

Remove one of those fillings in a mass where decay has recurred, and examine it with a glass. In one case it is imperfect preparation; in another, imperfect packing; in another, injury to the walls in packing; in another, it is not properly finished. Now, all these imperfections can be avoided, and should be, rather than resort to the new method.

Another source of failure of fillings in this latitude, still more prolific than "uncleanness," is a lack of full and complete restoration of the crowns, even in what are called "contour fillings." The space at the cervix is contracted by the teeth coming together, producing soreness, and, by reason of the tenderness of parts and the lessened space,

*preventing* cleanliness. In this manner we incur to some extent the evils of the "cut away" plan. We establish a set of circumstances calculated to result in decay; whereas, if there is full restoration, there is less probability of decay than in the original condition, for the simple reason that the point where it has occurred, and generally does occur is now occupied by an undecaying substance. It did not in that particular case originally, and does not generally, commence to decay at any part of the line now occupied by the margin of the filling. The simple fact that decay recurs is sufficient evidence of an imperfect operation, or the result of the cut-away plan and coming together again.

But Prof. Arthur requires even for the success of his plan that the tape must be used "several times each day" (p. 159) and "carefully examined at short intervals" (p. 157). Have not we reason to believe that with *such* treatment decay would *never* occur, even on the proximate surfaces of bicuspids and molars?

On page 95 we are told that it not the "slightest disfiguration" to cut away the incisors. A clerical friend, whose eye lit upon the proposition, remarked, "What a pity the Lord did not know about that in the first place."

These teeth are provided by nature with a thicker, heavier portion of enamel, and especially the linguo-proximate portion, often somewhat in the form of a scroll, with more or less of a depression in the centre, or between the two enlarged portions of enamel. The distance between these two extreme points is as great or greater than between the two points immediately anterior, often a little depressed, being the exact points where decay is expected to make its attack. Can we claim that there is no deformity when this mass of enamel is removed, which was placed at this particular point to sustain the cutting edges—which are very likely to be already checked, and so often break away—and to furnish a triturating surface in occlusion with the points and anterior surfaces of the inferior incisors, as important as the grinding surfaces of the molars? When the natural relations and conditions are changed it *is* a deformity, and the cutting away of the front teeth at these points is a deformity both seen and felt. The front teeth, as well as the bicuspids and molars, are formed to protect the gums. The gums will bear to be wounded but will not bear an unnatural, continued pressure.

On page 169 the author says, "What I propose is the separation of teeth closely in contact, which are of such frail character, and are exposed to such destructive influences, that their decay is inevitable, or has already occurred. This proposition may be met in this manner: 1. The care the author *requires in any case* as a *sine qua non*, will very generally answer the saving purpose without any operation. 2. The teeth are never in actual contact except at a small point—the most projecting part of the crown. 3. It is folly to remove the only points of contact when contact is necessary and inevitable. 4. Lessening the point of contact, while enlarging the space already too much at the cervix (the second V), will give all the advantage which can be *secured* by any means.

But our author speaks of leaving projecting dentine near the gum (p. 158). How are you going to get projecting dentine near the gum, and what will be the shape of your tooth by the time you get it? Where is the gum and the process by the time the dentine of the two teeth come in contact?

Again, how certain are we when we determine "that decay is inevitable." How often do we see cases where the "destructive influences" have been removed and the "frail character" no longer appears.

I have endeavored to state the reasons of my conviction, based upon theory and practice, that the plan of artificial separation of the teeth as proposed in this book is not necessary; is not the best treatment; and is a failure—*Dental Cosmos.*

